# The Law Relating to Infectious Diseases.—I

**Published:** 1902-07-19

**Authors:** J. E. R. Stephens

**Affiliations:** Barrister-At-Law, Author of "Digest of Public Health Cases," etc. etc.


### THE INSTITUTIONAL WORKSHOP.

### THE LAW RELATING TO INFECTIOUS DISEASES.—I.

By J. E. R. STEPHENS, Barrister-at-law, Author of "Digest of Public Health Cases," etc. etc.

THE law relating to infectious disease varies in different sanitary districts according as the sanitary authorities have adopted, or not, all or any of the sections of the Infectious Disease (Prevention) Act, 1890. It is left entirely to the option of every urban and rural sanitary authority whether they will adopt, and whether after adopting they will rescind, any of these sections. The Public Health Act, 1875, contains numerous provisions with regard to infectious diseases, and the steps to be taken by local authorities for preventing and checking the spread of such disorders. These authorities are empowered to see that houses and articles infected are properly cleansed and disinfected (sects. 120-123), and to make provision for the conveyance of patients suffering from such diseases to hospitals (sects. 123, 124); they may provide hospitals (sect. 131), and must carry out any regulations made by the Local Government Board under the power given to the Board by sect. 134 to make such, whenever any part of England appears to be threatened with, or is affected by, any formidable epidemic, endemic, or infectious disease. Local authorities are also required to make by-laws for common lodging-houses, dealing, *inter alia*, with the notification of any case of infectious disease occurring therein, and the taking of precautions against the spread of disease (sect. 80). By the same statute it is made an offence for a person while suffering from any such disorder wilfully to expose himself in a public place without taking proper precautions against spreading the disorder, or to enter a public conveyance without notifying the owner, conductor, or driver thereof that he is so suffering (sect. 126). It is also an offence for a person being in charge of anyone suffering from such a disorder to expose him in a public place without taking similar precautions. To expose such a person on the highway is also indictable at common law. A public conveyance in which a person suffering from any such disorder has been carried must be disinfected immediately afterwards (sect. 127). An inn, house, or room in which a person so suffering has been, must previously have been disinfected before being let (sect. 128); and it is an offence for a person to make any false statement with respect to such house or room (sect. 129).

Further provision is made on the subject by the Infectious Disease (Notification) Act, 1889, and the Infectious Disease (Prevention) Act, 1890. Both Acts are adoptive; the latter being adoptive either in whole or in part. The Act of 1889 defines "infectious disease" for the purpose of the Act as meaning any of the following diseases, namely, small-pox, cholera, diphtheria, membranous croup, erysipelas, scarlatina or scarlet fever, typhus, typhoid, enteric, relapsing, continued, or puerperal fevers, as well as any other infectious disease which by a resolution duly passed by the local authority and sanctioned by the Local Government Board is declared to be included in this the definition (sects. 6 and 7). The occurrence of any such disease in any building used for human habitation, or in any ship, boat, tent, van, shed, or similar structure (but not including Crown buildings) must be notified to the medical officer of health of the district (sects. 3, 13 and 15), the duty of giving this notice being cast primarily on the head of the house to which the patient belongs, failing whom the nearest relative present, or on his default on the occupier of the building (sect. 3). Any medical practitioner visiting such patient is also bound to give notice.

By the Act of 1890 provision is made for the inspection of dairies, and if necessary for the prohibition of the supply of milk from the same, where it appears the infectious disease is caused by the consumption of milk supplied therefrom (sect. 4). Provision is also made for the cleansing and disinfecting of premises, bedding, and other articles which have been infected (sects. 5 and 6). Penalties are imposed on persons ceasing to occupy houses or rooms in which six weeks previously any person suffering from any infectious disease has been, without previously disinfecting the same and all articles therein likely to retain infection, or without giving notice to the owner (sect. 7). Provision is likewise made for the speedy interment of the bodies of persons dying of infectious diseases, and for the disinfection of any public conveyance other than a hearse in which any such body has been carried (sects. 8-11). Orders may be made for the detention of infected persons who are without proper lodging (sect. 12), and temporary shelter may be provided for persons compelled to leave their dwellings in order that the same may be disinfected (sect. 15). Infectious rubbish thrown into ashpits must be disinfected (sect. 13). The chief provisions of these statutes have been reproduced in practically identical terms in the Public Health (London) Act, 1891 (sects. 55-74). By the Public Health Act, 1896, the Local Government Board may make regulations providing *inter alia* for signals being hoisted by vessels having any case of epidemic, endemic, or infectious disease on board, for the detention of vessels and persons on board, and the duties to be performed in cases of such disease by master pilots, and other persons on board.

By several statutes the Local Government Board is empowered to frame and issue regulations to sanitary authorities prescribing the measures to be taken in the event of the outbreak of any epidemic, endemic, or infectious disease. For efficiently executing the requirements of the regulations sanitary authorities are authorised to borrow money from the Public Works Loan Commissioners, and certain formalities in connection with the same may in urgent cases be dispensed with if the Local Government Board deems it advisable (Epidemic, etc., Act, 1882, sect. 2). Various regulations have been issued from time to time by the Local Government Board under these statutory powers.

By the Public Health Act, 1875, sect. 120, where any local authority are of opinion, on the certificate of their medical officer of health or of any other legally qualified medical practitioner, that the cleansing and disinfecting of any house or part thereof, and of any articles therein likely to retain infection, would tend to prevent or check infectious disease, it is the duty of such authority to give notice in writing to the owner or occupier of such house or part thereof requiring him to cleanse and disinfect such house or part thereof and articles within a time specified in such notice. If the person to whom notice is so given fails to comply therewith, he is liable to a penalty of not less than one shilling and not exceeding ten shillings for every day during which he continues to make default; and the local authority shall cause such house or part thereof and articles to be cleansed and disinfected, and may recover the expenses incurred from the owner or occupier in default in a summary manner. Where the owner or occupier of any such house or part thereof is from poverty or otherwise unable, in the opinion of the local authority, effectually to carry out the requirements of this section, such authority may, without enforcing such requirements on such owner or occupier, with his consent cleanse and disinfect such house or part thereof and articles, and defray the expenses thereof.

A similar power of requiring filthy or unwholesome premises to be purified on the certificate of the medical officer, or two medical practitioners, is given by sect. 46 of the Act of 1875. That section provides that where, on the certificate of the medical officer of health or of any two medical practitioners, it appears to any local authority that any house or part thereof is in such a filthy or unwholesome condition that the health of any person is affected or endangered thereby, or that the whitewashing, cleansing, or purifying of any house or part thereof would tend to prevent or check infectious disease, the local authority shall give notice in writing to the owner or occupier of such house or part thereof to whitewash, cleanse or purify the same, as the case may require. If the person to whom notice is so given fails to comply therewith within the time therein specified, he shall be liable to a penalty not exceeding 10s. for every day during which he continues to make default; and the local authority may, if they think fit, cause such house or part thereof to be whitewashed, cleansed, or purified, and may recover in a summary manner the expenses incurred by them in so doing from the person in default. The above provision extends to rural as well as urban authorities. It will be observed that by sect. 120 the certificate of one medical practitioner is sufficient. Local authorities are enabled by the Cleansing of Persons Act, 1897, to provide buildings, appliances, and attendants for cleansing free of charge the person and clothing of anyone who is infested with vermin.

If a nuisance causes a house or building to be unfit for human habitation, its use for that purpose may be prohibited under sect. 97 of the Act of 1875. If the medical officer of health finds that any dwelling-house in the district is in a state so dangerous or injurious to health as to be unfit for human habitation, he must report thereon to the local authority of the district. He is also to do so on receiving a representation from four or more householders living in or near the street where the premises are situated. The local authority are then to take action thereon in the manner provided by Part II. of the Housing of the Working Classes Act, 1890. In urban districts the medical officer of health may make an official representation to the local authority to the effect that an area is unhealthy, with a view to an improvement scheme being made for the rearrangement and reconstruction of the streets and houses in such area.

Where two convictions against the provisions of any Act relating to the overcrowding of a house have taken place within a period of three months (whether the persons convicted were or were not the same), a court of summary jurisdiction may, on the application of the local authority of the district in which the house is situated, direct the closing of the house for such period as the court may deem necessary (sect. 109 of the Act of 1875). Any house, or part of a house, so overcrowded as to be dangerous or injurious to the health of the inmates, whether or not members of the same family, is a nuisance, and may be dealt with summarily under sects. 93–96 of the Public Health Act, 1875.

Where the Infectious Disease (Prevention) Act, 1890, has been adopted, sect. 5 of that Act, which is substituted for sect. 120 of the Act of 1875, above referred to, provides for the notice to the owner or occupier being given by the clerk without referring to the local authority. It enacts that where the medical officer of health of any local authority or any other registered medical practitioner certifies that the cleansing and disinfecting of any house, or part thereof, and of any articles therein likely to retain infection, would tend to prevent or check infectious disease, the clerk to the local authority shall give notice in writing to the owner or occupier of such house or part thereof that the same and any such articles therein will be cleansed and disinfected by the local authority at the cost of such owner or occupier, unless he informs the local authority within 24 hours from the receipt of the notice that he will cleanse and disinfect the house or part thereof and any such articles therein to the satisfaction of the medical officer of health within a time fixed in the notice. If within 24 hours from the receipt of the notice the person to whom the notice is given does not inform the local authority as aforesaid, or if, having so informed the local authority, he fails to have the house or part thereof and any such articles disinfected within the time fixed in the notice, the house or part thereof and articles shall be cleansed and disinfected by the officers of the local authority under the superintendence of the medical officer of health, and the expenses incurred may be recovered from the owner or occupier in a summary manner. Provided that where the owner or occupier of any such house or part thereof is unable, in the opinion of the local authority or of their medical officer of health, effectually to cleanse and disinfect such house or part thereof, and any article therein likely to retain infection, the same may without any such notice being given, but with the consent of such owner or occupier, be cleansed and disinfected by the officers of and at the cost of the local authority. By sect. 17 of the same Act it is enacted that for the purpose of carrying into effect the provisions of the above section the local authority may, by any officer appointed in that behalf, who shall produce his authority in writing, enter on any premises between the hours of ten o'clock in the forenoon and six o'clock in the afternoon. The inability to cleanse, etc., need not arise from poverty; it may arise from want of proper appliances.

The superiority of the provisions of sect. 5 of the Act of 1890 over the corresponding provisions of sect. 120 of the Public Health Act, 1875, is very great. In the first place, they insure that the notice to disinfect shall be served on the owner or occupier immediately after the receipt by the clerk to the sanitary authority of the certificate of the medical officer of health or other medical practitioner. In the second place, they give the owner or occupier an option, which must be exercised within twenty-four hours, of determining whether he will do the disinfecting himself, or leave it to the sanitary authority to do it for him at his cost. In most cases the work of disinfecting can be done better and more cheaply by the officers of the sanitary authority than by private individuals; and in the interests of the public health, as well as those of the owner or occupier, it is desirable that it should be done by these officers. The Public Health Act, on the other hand, imposes a fine on any owner or occupier who leaves the work to be done by them. In the third place the Public Health Act merely requires the house, room, or articles to be disinfected, and in no way secures that the disinfection shall be effective. Sect. 5 of the Act of 1890, on the other hand, requires that the disinfection shall be to the satisfaction of the medical officer of health, and gives the officer of the sanitary authority power to enter the house for the purpose of carrying the section into effect.

(*To be continued.*)

